# Cellular Mechanisms of Inflammaging and Periodontal Disease

**DOI:** 10.3389/fdmed.2022.844865

**Published:** 2022-05-06

**Authors:** Daniel Clark, Allan Radaic, Yvonne Kapila

**Affiliations:** 1 Department of Periodontics and Preventive Dentistry, School of Dental Medicine, University of Pittsburgh, Pittsburgh, PA, United States; 2 Orofacial Sciences Department, School of Dentistry, University of California, San Francisco (UCSF), San Francisco, CA, United States

**Keywords:** inflammaging, periodontal disease, immune cells, aging, immune response

## Abstract

Increased age is associated with an increased prevalence of chronic inflammatory diseases and conditions. The term inflammaging has been used to describe the age-related changes to the immune response that results in a chronic and elevated inflammatory state that contributes, in part, to the increased prevalence of disease in older adults. Periodontal disease is a chronic inflammatory condition that affects the periodontium and increases in prevalence with age. To better understand the mechanisms that drive inflammaging, a broad body of research has focused on the pathological age-related changes to key cellular regulators of the immune response. This review will focus on our current understanding of how certain immune cells (neutrophils, macrophages, T cells) change with age and how such changes contribute to inflammaging and more specifically to periodontal disease.

## INTRODUCTION

Advances in medical care and technology have resulted in increased life expectancy. The populations of those over age 65 are expected to grow substantially within the United States and globally over the coming decades (1, 2). Despite the recent advances, there is still an increased burden of disease associated with increased age. Addressing the health care needs of this growing population will present challenges. A better understanding of how age contributes to disease susceptibility will allow us to better manage this unmet clinical need in older adult populations.

**FIGURE 1 ∣:**
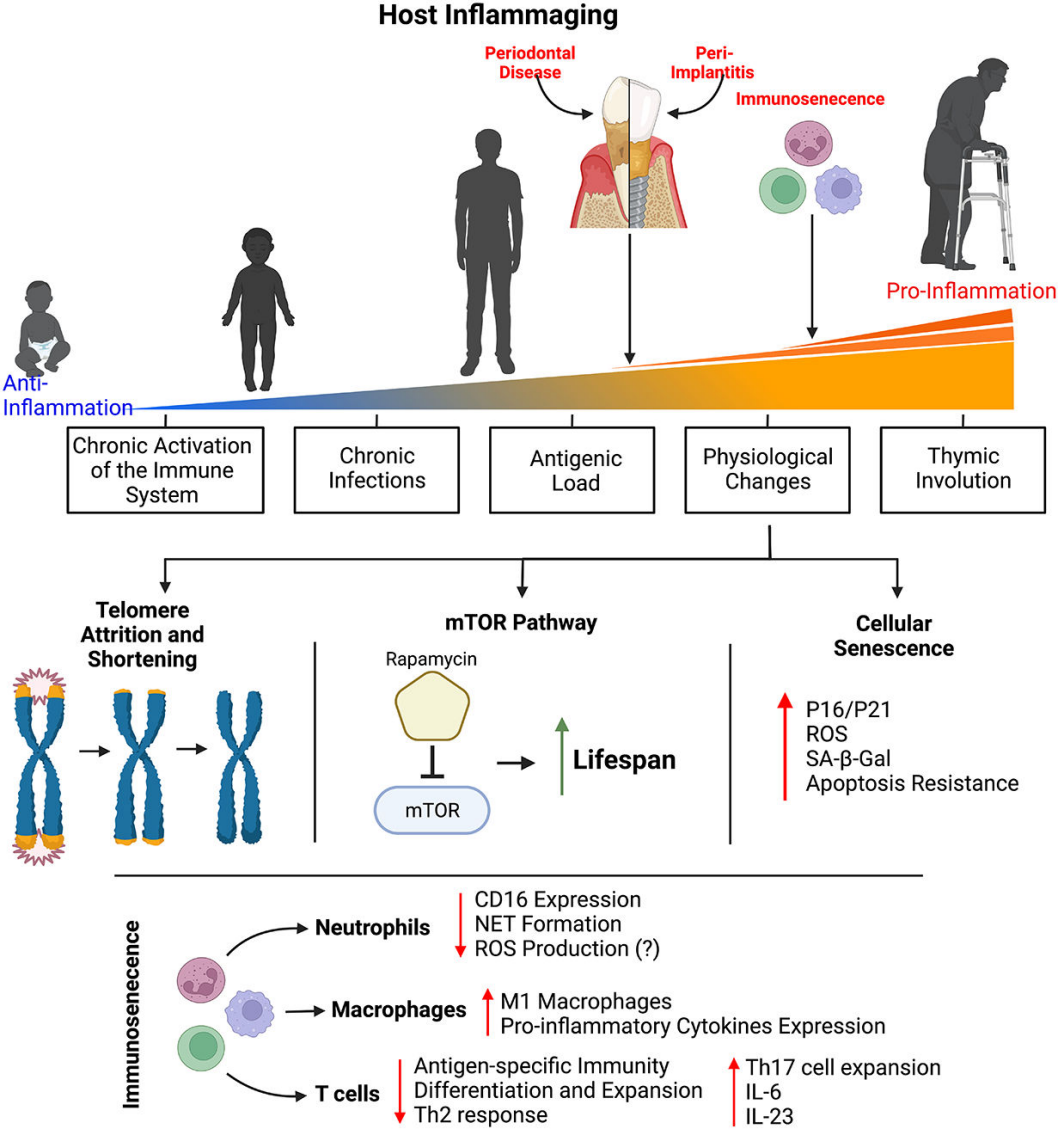
Physiological changes that occur with age have been shown to contribute to the myriad of disease that increase in prevalence with increasing age, including periodontal disease. Significant attention has been paid to better understand the pathogenic age-related changes that affect the immune system. Age-related immune dysregulation has generally been described as resulting in immunosenescence (a decreased ability to mount an effective immune response and a resulting immunocompromised status) or inflammaging (a chronically elevated and hyper-immune response). Immune cells with critical roles in regulating inflammation within the periodontium (neutrophils, macrophages, and T cell) have demonstrated age-related changes that contribute to immunosenescence and inflammaging. Beyond the immune system, other age-related changes that occur systemically have been shown to be pathogenic. Telomere attrition and shortening occurs throughout most cells and is highly associated with the onset of age-related disease. The mTOR signaling pathway is involved in a variety of basic cellular functions across cell types and inhibiting its activity results in significant gains in life span in animal models. Cellular senescence is a state of proliferation arrest is cells. Senescent cells accumulate in tissue with increasing age and produced a variety of pro-inflammatory cytokines, chemokines, and tissue remodeling enzymes, known as the senescent cell secretory phenotype (SASP), that contributes to age-related pathology.

Chronic diseases, such cardiovascular disease, type II diabetes, and Alzheimer’s disease and related dementias all increase in prevalence with increasing age. In addition, periodontal disease is one of the most common chronic diseases and the prevalence of periodontal disease also increases with age (3, 4). Much attention has been paid to how age-related physiological changes affect health span and lifespan (Figure 1). For example, telomere attrition occurs throughout mammalian aging and is associated with the onset of age-related disease (5). The mammalian target of rapamycin (mTOR) pathway is involved in a diverse set of cellular processes that generally control growth and homeostasis (6). Dysregulation of this pathway is strongly linked to age-related disease. Additionally, cellular senescence is a state of proliferation arrest is cells. Senescent cells accumulate in tissue with increased age and contribute to a variety of age-related disease, in part, through their characteristic secretion of pro-inflammatory cytokines, chemokines, and tissue remodeling enzymes (7).

The focus of this review will be on the age-related changes that affect the immune system and contribute to disease. The changes that occur to the immune system with age are quite diverse and differ across the adaptive and innate immune systems as well as across the different immune cell types (Figure 1). These pathological changes have generally been described as contributing to immunosenescence or inflammaging. Immunosenescence describes the age-related dysregulation of the immune response that results in immunocompromise and contributes to increased disease susceptibility (8). This immunocompromised status is associated with higher rates of infection and the resulting increased morbidity and mortality in older adults (8, 9). Inflammaging describes a chronically elevated and dysregulated inflammatory response that occurs with increasing age (10). Even in otherwise healthy older adults, there are increased levels of circulating pro-inflammatory factors such as interleukin-6 (IL-6), tumor necrosis factor α (TNFα), and C-reactive protein (CRP) compared to young adults (11, 12). Inflammaging is associated with a chronic inflammatory component that underlies many of the age-related diseases.

Given the current understanding of periodontal disease pathogenesis, immunosenescence and inflammaging may both be important factors contributing to the increased prevalence of periodontal disease in older adults. Within the periodontium, a strict regulation of the host inflammatory response to the oral microbes that inhabit these tissues is required to maintain periodontal health. As a result of immunosenescence, there may be a decreased ability to mount an effective immune response and adequately clear the invading oral pathogens. Similarly, the effects of inflammaging may result in a hyperreaction to the inflammatory stimuli resulting in collateral damage to the periodontal tissue as well.

The cause of inflammaging and immunosenescence is likely multifactorial. This review will examine the current understanding of age-related changes that affect neutrophils, macrophages, and T cells. These cells have been chosen here due to their known involvement in the pathogenesis of periodontal disease and their involvement throughout the initiation, propagation, and resolution of inflammation. It is also important to appreciate that there are many other factors that contribute to age-related immune dysregulation but are outside the scope of this review. For instance, in addition to neutrophils, macrophages and T cells, many other immune cells have been implicated in periodontal disease pathogenesis, including dendritic cells, B cells and natural killer T cells (13). Each of these cells have also demonstrated pathologic age-related changes but they will not be discussed in depth here.

This review will provide a brief explanation of the normal function of neutrophils, macrophages and T cells during infection and regulation of inflammation. The current understanding of how such functions change with age will be presented, highlighting how these age-related changes can be pathogenic in periodontal disease. A better understanding of the pathogenic age-related changes to these immune cells during periodontal disease may lead to improved therapeutic targets and better options for managing disease in older adult populations.

## NEUTROPHILS

Neutrophils are present within the periodontium in health and disease. Recruitment of neutrophils locally to the periodontium is initiated by a cascade of chemotactic signals from oral microorganisms (14). At the site of inflammation, neutrophils will exit the microvasculature, enter the gingival tissues, and continue their migration toward the gingival crevice where there is a high number of microorganisms and their associated chemoattractants. Even during periods of clinical health, the presence of neutrophils within healthy tissue has been demonstrated, as they can be recruited by the oral microorganisms constantly present at the gingival crevice (15, 16). Periodontal disease is characterized by a large increase in the number of neutrophils within the periodontium and the gingival crevice. Disease results in a significant increase in chemoattractant signals that are both endogenous, such as pro-inflammatory signals derived from the epithelia, and exogenous, such as LPS derived from the local bacterial plaque that cause the influx of neutrophils locally (14).

Health and disease are characterized, in part, by a difference in the number of neutrophils locally present. Therefore, it is of interest to understand if age affects the number of neutrophils within the tissue. Studies have measured the number of circulating monocytes as well the number of their progenitors within the bone marrow and have found no difference as a function of age (17, 18). Other groups have shown that the ability of neutrophils to respond to chemoattractants and migrate to the site of inflammation appears to remain intact with increased age (19, 20) or demonstrate a slight reduction in response (21, 22). *In vitro* investigations of the chemotactic response have shown that neutrophils from older adult subjects demonstrate a reduced response to granulocyte-colony stimulating factor (G-CSF) and N-formyl-Met-Leu-Phe (FMLP) peptide (18, 23). However, the chemotactic response to granulocyte macrophage-colony stimulating factor (GM-CSF) and IL-3 were not affected by age (18). Similarly, the molecules that promote the migration of neutrophils out of the vessels and into tissue, such as CD15, CD11a and CD11b, do not change as a function of age or are slightly increased (20, 24, 25). Taken together, the small changes that have been reported are unlikely to result in an inadequate quantity of neutrophils that respond to infection in older adults, including in response to the oral microorganisms present with periodontal disease (26).

The number of neutrophils able to respond to infection appears to remain largely intact in aging populations. However, age-related changes that affect neutrophil function, especially antimicrobial activity, may be more pathologic. Upon infiltration into the tissue, neutrophils are equipped with multiple antimicrobial strategies to reduce the microbial load. Phagocytosis describes the neutrophil’s ability to ingest and kill microbes intracellularly, and studies have shown that phagocytosis by neutrophils is attenuated in older adults (27, 28). Multiple mechanisms for the age-related decrease in phagocytosis have been proposed. Neutrophils from older subjects demonstrated decreased expression of CD16 (29). CD16 is a Fcγ receptor expressed on neutrophils that recognizes IgG-opsonized microorganisms and facilitates their engulfment (30). Others have similarly suggested that neutrophil recognition by antibodies and complement is attenuated in older populations, which contributes to the age-related decrease in phagocytosis (20, 25).

Neutrophils are also capable of producing cytotoxic reactive oxygen species (ROS) as an antimicrobial defense mechanism. Neutrophils release ROS intracellularly to kill phagocytized microbes or extracellularly to kill microbes within the tissue (31). Some studies have shown that the production and release of ROS was decreased in neutrophils from older groups compared to young (21, 25, 32). In contradiction, others have reported that the ROS production by neutrophils is increased in samples from older adults compared to young (33, 34). However, the increased ROS production was associated with increased level of circulating pro-inflammatory cytokines in older adults. Thus, it is not clear if the age-related changes in ROS production are a result of intrinsic changes to neutrophils from older adults or a result of an increased presence of pro-inflammatory stimuli. Additionally, the attenuation of ROS production as a function of age may be pathway or stimulus specific. One study demonstrated that ROS production by neutrophils from old donors was decreased when stimulated by *Staphylococcus aureus* but there was no change in ROS production when old neutrophils were stimulated by *Escherichia coli* compared to young (28).

Another antimicrobial strategy utilized by neutrophils is the formation of neutrophil extracellular traps (NETs). In response to certain microbial stimuli, neutrophils will undergo a form of cell death known as NETosis, where the nuclear and plasma membrane disintegrate and a decondensed DNA structure is released extracellularly forming the physical NET (35). NET formation in the extracellular space provides a physical barrier to trap microorganisms from further invasion, and also contains antimicrobial agents, including neutrophil elastase, cathepsin G, and myeloperoxidase, to kill microorganisms and eliminate their associated virulence factors (36). NET formation appears to be an important host defense mechanism during periodontal disease (37). Hirschfield and colleagues demonstrated that NET formation was stimulated by 19 different microbial species involved in periodontal disease (38). Similar to other antimicrobial strategies utilized by neutrophils, NET formation also appears to be attenuated with increased age. Multiple *in vitro* studies have demonstrated decreased NET formation by neutrophils from old subjects compared to young (39-41). Research using mouse models of *Staphylococcus aureus* infection have demonstrated that NET formation was attenuated in old mice which was associated with increased dissemination and more severe infection compared to young (41).

In summary, increased age has a pathological effect on the antimicrobial function of neutrophils and less of an effect on the number of neutrophils recruited locally. This attenuation in antimicrobial activity likely contributes to the increased prevalence of periodontal disease in older adults, as has been shown in other age-related disease (42). The age-related decrease in phagocytosis or NET formation by neutrophils could result in a decreased ability to kill and clear pathogenic microbiota within the periodontium and an associated prolonged inflammatory response. Inadequate resolution of inflammation can propagate the tissue destructive mechanisms that contribute to the clinical hallmarks of periodontal disease (43). However, it is also important to appreciate that the same antimicrobial actions of neutrophils that are toxic to microbes are also toxic to host cells (43). This points to the importance of a tightly regulated immune response within the periodontium and how perturbations of the response can lead to disease. More evidence is needed to understand the specific effects of these age-related changes to periodontal disease susceptibility.

## MACROPHAGE

The two general classes of macrophages are tissues resident macrophages and circulating monocyte-derived macrophages. Circulating monocyte-derived macrophages appear in tissue in response to infection or injury. Circulating monocytes are recruited locally where they differentiate into macrophages and migrate into tissue (44). Within the periodontium, macrophages are rapidly present in response to invading oral pathogens and their associated chemoattract signals. Macrophages demonstrate multiple antimicrobial strategies to eliminate invading microbes and are able to both propagate or resolve the inflammatory response through the secretion of cytokines and chemokines (44, 45). Tissue resident macrophages are present steadily within tissue and arise developmentally from the yolk sac, which is distinct from the hematopoietic origin of monoctyes (46). Tissue resident macrophages are an emerging area of interest that have been characterized in a variety of tissues, such as bone, central nervus system, and liver. They exhibit a heterogenous set of functions including involvement in homeostasis and regeneration of tissue that appears to be unique to a given tissue (46). However, to date, no tissue resident populations of macrophages within the periodontium have been identified.

Phagocytosis is an important host defense mechanism exhibited by macrophages, which involves detection, ingestion, and killing of foreign material (47). Macrophages also ingest and degrade apoptotic cells, cellular debris, and damaged tissue after infection and injury or as part of normal homeostatic functions within the tissue (48). Macrophages have a repertoire of Toll-like receptors and pattern recognition receptors to detect necrotic and injured tissue or foreign LPS from invading microbes in order to sense and respond appropriately (49, 50). Macrophages specifically respond to oral periodontal pathogens. When stimulated with *Porphyromonas gingivalis* and *Aggregatibacter actinomycetemcomitans*, macrophages demonstrated an inflammation propagating response via the production of pro-inflammatory cytokines (45). Interestingly, the macrophages demonstrated an ability to mount a unique response to the individual pathogen, with *A. actinomycetemcomitans* stimulating increased expression of chemokines that promote T cell recruitment. In the presence of increased microbial infection, macrophages act to elevate the immune response. Macrophages release pro-inflammatory cytokines (iNOS, TNFα, IL-1β, IL-6) that propagate further immune cell recruitment (51). To further elevate the immune response, macrophages also function as antigen presenting cells to stimulate T cell activity and promote an adaptive immune response (45). T cell recruitment is a characteristic immune response during periodontal disease as discussed in the following section.

During the active resolution of infection, macrophages also act to downregulate the inflammatory response and promote tissue repair. Macrophages resolve inflammation through the production of arginase and anti-inflammatory cytokine IL-10 and promote healing of damaged tissues through the production of growth factors (TGFβ, VEGF) (52, 53). The anti-inflammatory and pro-inflammatory activities of the macrophage have generally been defined along a spectrum of M1 (pro-inflammatory) and M2 (anti-inflammatory) macrophage phenotypes. The understanding of macrophage phenotypes is continually evolving and beyond the scope of this manuscript. Therefore, the M1 and M2 phenotype designation will only be used to generally describe a pro-inflammatory and anti-inflammatory phenotype without reference to the multitude of diverse phenotypes continuing to emerge in the field.

It is important to appreciate that the antimicrobial and immune propagating functions of M1 macrophages are part of a necessary immune response to effectively defend against microbial infections. However, the same functions have also been implicated in the pathogenesis of periodontal disease where prolonged pro-inflammatory processes ultimately lead to tissue destruction. M1 macrophages have been identified in gingival samples of humans with periodontal disease at higher levels compared to healthy gingival samples. The increased M1 macrophage numbers were also associated with the presence of increased inflammatory cytokines in the gingiva (54). Similarly, other studies showed that an increase in M1 macrophages was associated with increased severity of periodontal disease whereas an increase in M2 macrophages was associated with decreased disease severity (55, 56). Proper regulation of the host response involves the appropriate transition from the M1 to M2 macrophage phenotypes to resolve inflammation and to minimize disease severity. Perturbations of macrophage phenotype as a result of systemic disease or increased age may contribute to the pathogenesis of many inflammatory conditions, including periodontal disease.

The effect of age on macrophages is of significant interest and studies have evaluated the number of macrophages that locally respond to infection or injury as a function of age. In a mouse model of periodontal disease, the number of macrophages that were present in the periodontium was similar in old and young mice during health, during periodontal disease, and during disease recovery (57). Others have similarly shown no difference in macrophage number as a function of age within muscle (58), or in the periotoeneum (59). Conversely, decreased numbers of macrophages in the lungs of older humans and mice has been demonstrated (60, 61).

Recent evidence has suggested that macrophage activity and the associated phenotypes may be detrimentally affected by age and such age-related changes may be pathogenic in disease including periodontal disease (62). However, when using *in vitro* study designs, age-related changes to macrophage activity have demonstrated conflicting results. Studies evaluating cytokine production by macrophages isolated from old animals and humans have shown increased (63), decreased (64), or no change (65) in pro-inflammatory cytokine production compared to young controls. Similarly, the phagocytic activity of macrophages was shown to be increased (66), decreased (59), or not changed (67) as a function of increased age. These conflicting results may be related to the differences in the *in vitro* cell culture environment compared to the *in vivo* environment. Aging is associated with changes in the bone marrow microenvironment, the progenitor cell source of monocyte-derived macrophages (68). Aging is also associated with higher levels of circulating pro-inflammatory cytokines, which may have an effect on the circulating monocytes before being recruited and differentiating into macrophages (11). Together, these environmental changes that occur with aging may have a significant effect on macrophage activity that is not readily replicated in the *in vitro* environment.

A better understanding of how age affects macrophages is now emerging with the use of next generation sequencing methodologies. One bulk RNA sequencing study isolated macrophages that had responded locally to an injury in bone in old and young mice (69). Macrophages from old mice were transcriptionally distinct from the macrophages from young mice and demonstrated increased expression of pro-inflammatory cytokines and other genes involved in the propagation of inflammation compared to young (69). Another study using single cell RNA sequencing similarly showed that alveolar lung macrophages from old mice demonstrated increased pro-inflammatory gene expression compared to the young samples (70). Interestingly, these age-related transcriptional changes are not consistent across all immune cell types. A study using single cell RNA sequencing to analyze immune cells of old and young mice demonstrated that some cell types showed small age-related changes whereas others, including macrophages, demonstrated a unique aging transcriptional profile (71).

Studies that have attempted to directly implicate age-related changes in the macrophage with the pathogenesis of periodontal disease have been limited. A recent study showed that depleting macrophages in old mice enhanced the recovery from periodontal disease after the ligature was removed, with improved resolution of inflammation and decreased disease severity compared to untreated old mice (57). Interestingly, depletion of macrophages in young mice had no measurable effect on disease recovery. These findings may suggest that the age-related defect in macrophage activity is focused on the resolution of inflammation. Another study similarly demonstrated the burden of macrophages in old animals, by showing that depletion of macrophages in old mice improved fracture healing outcomes compared to non-treated controls (69). Old animals exhibit delays in fracture healing and cutaneous wound healing, and studies have demonstrated that the healing could be similarly improved in old animals when macrophages from young mice were transplanted into the old mice (72, 73).

In summary, these studies demonstrate that macrophages undergo age-related changes that likely contribute to the pathogenesis of periodontal disease. Significant work within the periodontal and wider medical fields has supported the importance of proper regulation of macrophage phenotype in the prevention of inflammatory-related disease. The emerging next generation sequencing methodologies are providing a more accurate assessment of the *in vivo* phenotype, and such findings support the conclusion that macrophage phenotypes are affected by age and assume a more pro-inflammatory phenotype that may contribute to inflammaging. In the treatment of periodontal disease, the removal of the bacterial inflammatory stimuli results in an active shift toward resolution of inflammation driven, in part, by macrophages. Thus, an age-related delay or perturbation in the switch to M2-driven resolution of inflammation would result in the continued propagation of the osteolytic processes driven by M1 macrophages, or their downstream mediators, and an increase in disease severity.

## T CELLS

The innate immune response is responsible for the initial host defense to microbial pathogens within the periodontium. The prolonged presence of pathogens and the sustained innate immune response will eventually illicit an adaptive immune response. T cells are an important driver of the adaptive immune response and demonstrate a heterogenous repertoire that can recognize a broad range of antigens. Naïve T cells will interact with antigen presenting cells, such as dendritic cells, macrophages, Langerhans cells and B cells, that will promote their differentiation toward a variety of T cell subpopulations that are equipped to mount a more specific immune response (74). Many T cell subpopulations have been identified and shown to be important regulators of health or disease. The discussion below is limited to the T cell subpopulations that have previously been characterized within the periodontium.

CD4+ T helper cells are the most abundant T cell subpopulation found in gingiva (75). They are generally further characterized as Th1 or Th2 subsets. Th1 subsets demonstrate a pro-inflammatory phenotype that produce a variety of cytokines and chemokines that can illicit osteolytic processes during periodontal disease (76-78). The Th2 subsets contribute to an anti-inflammatory immune response to resolve inflammation or limit the tissue destructive proccesses (76, 77). Th2 cells act to resolve inflammation by secreting an anti-inflammatory cytokine profile and by directing B cell activity. Within the periodontium, Th2 cells activate and promote the expansion of B cell subsets that produce antibodies against oral pathogens (55). Diverse B cell subsets are present in periodontal heath versus disease. Thus, T cell regulation of B cell subsets is an important function to maintain periodontal health.

Th1 and Th2 subsets are both present in the gingiva during health and disease, with a shift toward an increased ratio of Th1 over Th2 cells occurring during periodontal disease (78). A further differentiated class of T cells, CD8+ cytotoxic T lymphocytes, also demonstrate a pro-inflammatory phenotype and are equipped to kill foreign or infected cells. Cytotoxic T lymphocytes produce antimicrobial cytokines (TNF-α and IFN-γ), secrete cytotoxic granules (perforin and granzymes) and can promote apoptosis of infected host cells (79). CD8+ cytotoxic T lymphocytes are also found in healthy gingival samples and increase in quantity during periodontal disease; however, their pathogenic contribution to periodontal disease is not fully understood (80). Regulatory T cell (Tregs) are a subpopulation believed to have important homeostatic roles within tissue. A body of work has focused on CD4+ Tregs; however, emerging evidence has supported the presence of CD8+ Treg populations with similar protective and homeostatic actions (80). Tregs have anti-inflammatory properties, in part through the production of IL-10 and TGF-β, that counteract the pro-inflammatory properties of the other T cell subpopulations (81). Tregs are protective of alveolar bone loss and can minimize osteolytic processes even in the presence of gingival inflammation (82).

Th17 cells are a subset of Th1 cells and secrete IL-17 in the promotion of inflammation (83). They have been implicated in the pathogenesis of periodontal disease due to IL-17 promotion of osteoclastic activity (83). Expansion of Th17 cell populations and the associated increase in IL-17 expression are characteristic of the pathogenic response in periodontal disease (84, 85). While multiple cells secrete IL-17, during periodontal disease Th17 cells represent 80% of the IL-17+ cells, which makes them a significant contributor to the pathogenesis of periodontal disease (84). Studies have shown that by inhibiting Th17 cell differentiation in mice, periodontal disease severity was decreased (84).

As discussed here, the multiple subsets of T cells contribute to the promotion or resolution of inflammation, but both were found to be present with the periodontium in health and disease. This demonstrates an ability of the oral microbiota to illicit an adaptive immune response even in conditions of health. It also supports the need for a precise balance of pro- and anti-inflammatory activities to properly respond to the microorganisms and to limit peripheral tissue damage that would contribute to periodontal disease. Therefore, it is of interest to try to understand how age affects the T cell activity

Age-related changes to T cells have been implicated in contributing to immunosenescence. The thymus is the site of naïve T cell production and, with increasing age, an involution of the thymus is observed (86). A reduction in the quantity of naïve T cells results in a decline of antigen-specific immunity and a resulting increased susceptibility to infection (86, 87). Further, age-related changes affecting the T cell antigen-receptors (TCR) may further limit the expansion and differentiation of T cells. Signaling through TCR in naïve T cells promotes the initial clonal proliferation in response to a specific antigen (88). With increased age, decreased signaling through the TCR or decreased sensitivity to TCR ligation was demonstrated (89). In addition, there is evidence of an age-related decrease in co-stimulatory receptors (CD27, CD28) that are expressed along with TCR in the differentiation of T cells (90). Together, the evidence supports that age results in a decrease in T cell antigen specificity. It could be expected that a decreased ability to mount a specific T cell response to oral pathogens may lead to advanced infection within the periodontium, and this age-related immunocompromised status may be a pathogenic contributor to periodontal disease.

Other age-related changes to T cells appear to result in an up regulation of an inflammatory response that could also contribute to disease pathogenesis. Age-related changes to CD4+ T helper cells have been reported to affect their responsiveness to receptor stimulation, resulting in a decreased Th2 response and a preference toward Th1 differentiation (91, 92). The increased Th1 response is associated with the increased pro-inflammatory cytokines that may drive, in part, the inflammaging phenotype associated with increased age. In addition, the higher ratio of Th1 cells over Th2 cells was found to be associated with increased alveolar bone loss, which may also be a contributor to the increased prevalence of periodontal disease with increased age (78). The Th17 cell population also demonstrates age-related changes that may contribute to a higher prevalence of periodontal disease. As discussed above, Th17 cells are potent pathogenic mediators in periodontal disease (83). Interestingly, with increased age, homeostatic expansion of Th17 cells within the gingiva was increased in old mice compared to young (93). Similarly, in human subjects higher circulating levels of Th17 cells were found in old subjects compared to young (94). It is not fully understood what drives the age-related increase in Th17 cell expansion. One possible explanation is that there is an age-related increase in the cytokines that promote Th17 cell expansion, IL-6 and IL-23 (95). While there are multiple sources of IL-6 and IL-23 secretion during disease, the M1 macrophage phenotype demonstrates increased expression of both cytokines during the propagation of inflammation (95). The previous section described an age-related shift in macrophage phenotypes toward M1. Interestingly, this age-related shift in macrophage phenotype may further drive a dysregulated inflammatory response by promoting Th17 cell expansion. More work is needed to understand how age-related changes may drive pathogenic interactions amongst immune cells in the promotion of periodontal disease.

## FUTURE DIRECTIONS AND CONCLUSION

Proper regulation of the host immune response is critical in maintaining periodontal health. As discussed here, age-related changes affecting neutrophils, macrophages, and T cells appear to promote a pathogenic immune response and contribute to the increased prevalence of periodontal disease in older adults. The burden of disease in older adults is well appreciated across dentistry and medicine. The field of geroscience works to understand how age-related physiological changes contribute to diseases that impact older adults. An improvement in our understanding of the basic cellular and molecular changes that occur with age will translate to a more targeted and individualized clinical care that focuses on the specific altered physiology that occurs with increased age. Research has already been performed showing the benefit of targeting the age-related perturbations to the immune system to improve outcomes in aging populations. Indeed, replacing components of the immune system of young mice into old mice, either by bone marrow transfer or heterochronic parabiosis, has demonstrated significant benefit in many different disease and injury models including fracture healing, skeletal muscle regeneration, cognitive performance, and Alzheimer’s disease (73, 96-98). The macrophage has emerged as a promising therapeutic target to treat age-related diseases due to our increasing understanding of its pathogenic changes that occur with age (99). Depletion of macrophages during the recovery of periodontal disease or during the fracture healing in mice resulted in improved outcomes in old mice (57, 69). In addition, injecting young macrophages into old mice improved cutaneous wound repair (72). The beneficial effects of the drug metformin on reducing the effects of age-related physiological changes have been shown to be, in part, through limiting macrophage differentiation and activation (100). Translating this approach to target macrophages in humans has largely focused on the treatment of cancer, as macrophages have been shown to contribute to tumor initiation, infiltration, and metastasis (101). In summary, the potential benefit of targeting the age-related physiological changes to treat disease in older adults is promising. Further work is needed to arrive at a better understanding of the changes that occur to key cellular regulators of the immune response in order to develop effective therapeutic targets.
